# Haloalkalitolerant Nitrile‐Utilizing *Rhodococcus* Strains Promote the Biodegradation of Nitrile‐Butadiene Rubber

**DOI:** 10.1155/ijm/7513883

**Published:** 2026-07-26

**Authors:** G. A. Syrovatskaya, A. Yu. Maksimov, O. V. Korotchenkova, Yu. G. Maksimova

**Affiliations:** ^1^ Laboratory of Molecular Biotechnology, Institute of Ecology and Genetics of Microorganisms Ural Branch Russian Academy of Sciences, Perm Federal Research Center, Perm, Russia; ^2^ Department of Microbiology and Immunology, Perm State University, Perm, Russia, psu.ru; ^3^ Laboratory of Geology of Mineral Deposits, Mining Institute Ural Branch Russian Academy of Sciences, Perm Federal Research Center, Perm, Russia

**Keywords:** biofilms, microplastics, nitrile-butadiene rubber (NBR), nitrile hydratase, nitrile-utilizing bacteria, *Rhodococcus*

## Abstract

Environmental pollution from polymers is reaching alarming levels. Nitrile‐butadiene rubber (NBR), widely used in medical glove manufacturing, contributes significantly to plastic and microplastic pollution. Nitrile gloves have become a major component of COVID‐19 pandemic‐related waste. In this study, we demonstrated the ability of haloalkalitolerant, nitrile‐utilizing *Rhodococcus qingshengii* IEGM 1416 and *R. erythropolis* IEGM 1417 to grow on a mineral medium containing NBR as the sole nitrogen source. Scanning electron microscopy and colony‐forming unit (CFU) counting confirmed the development of *Rhodococcus* biofilms on this material, representing the first step in polymer biodegradation. After UV and freeze‐thaw pretreatment of NBR, biofilm biomass for *R. qingshengii* IEGM 1416 and *R. erythropolis* IEGM 1417 reached (7.28 ± 0.48) × 10^6^ and (6.27 ± 0.72) × 10^6^ CFU/cm^2^, respectively. Using attenuated total reflectance Fourier transform infrared (ATR‐FTIR) spectroscopy, we identified structural changes in NBR following UV/freeze‐thaw treatment and subsequent 1‐month cultivation. The appearance of amide groups in the polymer structure resulted from the transformation of nitrile groups by bacterial nitrile hydratases. Oxidative aging of the material was observed after both UV irradiation and bacterial growth; notably, the effect varied between the different sides of the NBR due to structural heterogeneity. Enzymes from haloalkalitolerant nitrile‐utilizing rhodococci induce chemical changes in NBR, corresponding to the initial stages of backbone oxidation and nitrile group transformation.

## 1. Introduction

Environmental pollution by polymers is reaching a massive scale. Synthetic polymers are difficult to biodegrade, leading to the accumulation and long‐term persistence of these materials in the environment. Their partial degradation under the influence of physicochemical environmental factors gives rise to a new problem associated with microplastics [1, 2]. Therefore, there is a pressing need to study the ability of microorganisms to biodegrade synthetic polymers [3]. This task is complicated by the prolonged duration of the process and the difficulty of recording and analyzing changes occurring in the plastic. It requires the identification and selection of microorganisms that are potential degraders of synthetic polymers, as well as the study of metabolic pathways that can lead to polymer degradation. Attenuated total reflectance Fourier transform infrared (ATR‐FTIR) and Raman spectroscopy, liquid chromatography of released products, and a number of other methods are used to confirm plastic biodegradation [4].

Various synthetic polymers can be degraded by a heterogeneous group of enzymes, including lipases, esterases, laccases, amidases, cutinases, carboxylesterases, monooxygenases, dioxygenases, and others. Enzymes such as monooxygenases are responsible for dealkylation, epoxidation, hydroxylation, dehalogenation, deamination, desulfurization, sulfoxidation, and N‐oxide reduction reactions [5]. A common mechanism of oxidative cleavage has been proposed for all types of rubber‐degrading organisms. Since the double bonds of poly(cis‐1,4‐isoprene) are cleaved by oxygen incorporation, the reaction can be catalyzed by monooxygenase or dioxygenase [6]. Extracellular hydrolytic enzymes, such as hydrolases and other nonspecific enzymes such as peroxidases, laccases, and lignin peroxidases, also have the ability to degrade microplastics. Hydrolases can degrade the polymer surface, making it more susceptible to degradation by other enzymes, whereas laccase depolymerizes these materials through oxidative cleavage, causing the formation of microcracks on the surface [5].

Nitrile‐butadiene rubber (NBR) is a material used to manufacture disposable medical gloves. Furthermore, due to its exceptional resistance to oil, abrasion, and corrosion, NBR is widely utilized in the petrochemical, aerospace, and automotive industries [7]. The high demand for this product and its significant consumption volumes are causing an increase in the amount of NBR waste. Nitrile gloves have become a major component of COVID‐19 pandemic‐related waste. Moreover, NBR composites are being synthesized with the addition of other materials, particularly graphene, and ionic crosslinking agents to improve their wear resistance [8–10]. The downside of this improvement may be a reduction in their biodegradability, rendering NBR a persistent environmental pollutant.

There is limited information on the biodegradability of NBR and the effect of microorganisms on this polymer. Delgado‐Nungaray et al. reported the ability of *Pseudomonas aeruginosa* to grow on nitrile gloves [11]. *P. aeruginosa* was cultured in LB medium with nitrile gloves and biofilm formation on NBR and changes occurring in the polymer structure were demonstrated. Chiba et al. studied the degradation of the NBR carbon backbone by *Gordonia* sp. strain J1A and demonstrated that membrane‐bound nitrile rubber oxygenase (Nro1) is the key enzyme catalyzing the initial step of the NBR degradation reaction [12]. The mechanism underlying the degradation of NBR, particularly involving nitrile/amide‐splitting enzymes, is not yet fully understood [13]. Furthermore, the combination of bacterial action and the physical factors to which polymers may be exposed in the environment has not been sufficiently studied. In regions with temperate climates and seasonal cycling, waste in landfills is exposed to factors such as UV radiation and freeze‐thaw cycling. *P. aeruginosa*, considered by Delgado‐Nungaray et al. as a possible microbial degrader of this polymer [11], is an opportunistic pathogen that causes nosocomial infections and diseases in debilitated patients. Its potential virulence and multidrug resistance complicate its use as a biological agent for waste biodegradation [14].

Nitrile‐utilizing microorganisms have the ability to either transform nitriles into carboxylic acids in a single step via nitrilase (EC 3.5.5.1) or to catalyze a two‐step transformation: first by nitrile hydratase (EC 4.2.1.84) to amides and by amidase (EC 3.5.1.4) to carboxylic acids [15]. This suggests that microorganisms producing nitrile hydrolysis enzymes can participate in the transformation of the acrylonitrile component of NBR. However, it is unclear how their enzymatic systems will affect the acrylonitrile polymer, since these enzymes are intracellular and the polymers cannot penetrate the cell membrane.

Polymer degradation is known to be closely linked to biofilm formation, which is a community of microorganisms embedded in a matrix of extracellular polymeric substances (EPS). Microorganisms involved in microplastic degradation initially attach to their surface. The extent of the bacterial colonization of hydrophobic substrates largely depends on the hydrophobicity of the cell surface [16].

Extremophilic and extremotolerant microorganisms, particularly haloalkaliphilic (and haloalkalitolerant) microorganisms, possess great potential for the biodegradation of various xenobiotics and pollutants. They can grow under extreme conditions, such as those found in alkaline wastewater and saline soils. Such conditions can also be artificially created to accelerate degradation processes. Previously, we isolated *Rhodococcus qingshengii* IEGM 1416 and *R. erythropolis* IEGM 1417—nitrile‐utilizing strains containing a nitrile hydratase‐amidase enzymatic system, from the highly mineralized alkaline environment of a soda sludge storage facility [17]. In this study, we examined the ability of these two *Rhodococcus* strains, *R. qingshengii* IEGM 1416 and *R. erythropolis* IEGM 1417, to grow in a mineral medium with NBR as the sole nitrogen source. Scanning electron microscopy and colony‐forming units (CFU) counting were used to study the ability of rhodococci to form biofilms on the surface of this material. ATR‐FTIR spectroscopy was used to study changes in the NBR surface and determine the degree of its aging (oxidation), as well as the degree of transformation of the nitrile components of the polymer.

## 2. Materials and Methods

### 2.1. Bacterial Strains and Cultivation

*R. qingshengii* IEGM 1416 and *R. erythropolis* IEGM 1417, isolated from soda sludge with pH 11 and soil of the soda sludge storage facility with pH 8–8.5 [13], were cultivated on a mineral medium with the following composition (g/L): KH_2_PO_4_—1.0; K_2_HPO_4_ × 3H_2_O—3.7; NaCl—0.5; MgSO_4_ × 7H_2_O—0.5; FeSO_4_ × 7H_2_O—0.005; CoCl_2_ × 6H_2_O—0.01; pH 7.2 ± 0.2, with stirring at 120 rpm at 22°C–25°C. To select the optimal carbon source, glucose, sorbitol, dulcitol, inositol, mannitol, maltose, lactose, sucrose, citric acid, succinic acid, or acetic acid (each at 0.1%), or acetamide (50 mM) were added to the medium. Acetonitrile (9.12 mM) served as the nitrogen source. Mineral medium (2 mL) was added to each well of a sterile 24‐well plate and inoculated with 100 *μ*L of bacterial suspension (OD_540_ 0.5). Samples were taken after 24, 48, and 120 h, and the OD_540_ of the medium was determined on a Tecan plate reader (Infinite M1000, Switzerland) at a wavelength of 540 nm (certified linearity range of 0–2 OD, *R* ^2^ ≥ 0.999). To select the optimal nitrogen source for *R. qingshengii* IEGM 1416 and *R. erythropolis* IEGM 1417, NH_4_Cl/(NH_2_)_2_CO/NaNO_2_/NaNO_3_ (10 mM), or acetonitrile were added to a mineral medium containing sorbitol (1.5%) or glucose (1%) as a carbon source, respectively. Cultivation was carried out in flasks on a shaker at 120 rpm and 22°C for 10 days.

### 2.2. Determination of Nitrile Hydratase and Amidase Activity in *Rhodococcus* Strains

*R. qingshengii* IEGM 1416 and *R. erythropolis* IEGM 1417 were grown to stationary phase. The culture medium was centrifuged for 10 min at 4500 × g. The cells were washed with potassium phosphate buffer (pH 7.2) and centrifuged again. The cell pellet was suspended in 1 mL of phosphate buffer and supplemented with 0.3 M acrylonitrile to determine nitrile hydratase activity and 50 mM acrylamide to determine amidase activity. After 24 h of shaking at 25°C and 120 rpm, the reaction was quenched with 50 *μ*L of concentrated HCl and centrifuged for 10 min, and the reaction products (acrylamide and acrylic acid) were then analyzed in the supernatant. One unit of specific activity (U) was defined as the amount of biocatalyst forming 1 mmol of acrylic acid and/or acrylamide per hour. Nitrile hydratase and amidase activities were expressed as U/g.

Acrylamide, acrylic acid, and acrylonitrile in the reaction and culture media were determined by HPLC on an Infinity II LC 1260 chromatograph (Agilent, Germany) equipped with a Synergi 4u Hydro–RP 80A column (250 × 4.6 mm). The mobile phase consisted of 25 mM NaH_2_PO_4_ with 5% acetonitrile; the flow rate was 0.500 mL/min at 25°C, and detection was performed at 210 nm.

### 2.3. Growth of *Rhodococcus* Strains With NBR

*R. qingshengii* IEGM 1416 and *R. erythropolis* IEGM 1417 (OD_540_ 0.5–0.7) were inoculated into a nitrogen‐free mineral medium (pH 8) containing 1.5% sorbitol or 1% glucose, respectively. NBR, cut into 1 × 1 cm squares, was added as the sole nitrogen source at a ratio of 10 squares per 200 mL of medium. *Rhodococcus* strains were grown for 14 days at 25°C and 120 rpm on a shaker. Acrylamide, acrylic acid, and acrylonitrile concentrations in the culture medium were determined by HPLC after centrifugation and filtration through a 0.22 *μ*m syringe filter. The NBR samples were pretreated with an OUFd‐01 ultraviolet irradiator (“Solnyshko”, Russia) at a power of 300 VA from a distance of 10 cm for 15 min and/or subjected to three freeze‐thaw cycles (15 min each at –20°C/+25°C). Mineral medium without a nitrogen source served as a control. The number of CFU/mL in the culture media was determined by plating tenfold dilutions onto nutrient agar (“Biotekhnovatsiya,” Russia). Biofilm formation on the NBR was monitored by scanning electron microscopy.

*R. qingshengii* IEGM 1416 and *R. erythropolis* IEGM 1417 were precultured in mineral medium with 1.5% sorbitol and 50 mM acetamide as carbon sources, respectively, and 9.12 mM acetonitrile as a nitrogen source. After 10 days of cultivation, the biomass was harvested by centrifugation and washed. Then, 3.098 and 3.427 g of wet biomass (60% moisture content) of *R. qingshengii* IEGM 1416 and *R. erythropolis* IEGM 1417, respectively, were added to 200 mL of mineral medium containing 1.5% sorbitol or 1% glucose. No soluble nitrogen source was added; instead, 10 pieces of NBR (1 × 1 cm) were added per 200 mL of medium. The NBR was pretreated with UV and/or freeze‐thaw cycles. After 1 month of cultivation, the NBR squares were washed with phosphate buffer; ATR‐FTIR spectroscopy was then performed, and biofilms were visualized by scanning electron microscopy. To count the biofilm‐associated cells, the NBR was sonicated five times in an ultrasonic bath (“Elma,” Germany) in 1 mL of sterile 0.9% NaCl solution for 1 min with 2 min intervals. The CFU/mL was calculated after plating tenfold dilutions onto nutrient agar.

### 2.4. Scanning Electron Microscopy

Electron micrographs were obtained at accelerating voltages of 10–20 kV in the SE (secondary electron) mode using a VEGA 3 LMH scanning electron microscope (Tescan, Czech Republic) with an Oxford Instruments INCA Energy 250/X‐max 20 energy‐dispersive x‐ray microanalysis system. The NBR samples were predried in air at 25°C and sputter‐coated with carbon to improve electrical conductivity.

### 2.5. Fourier Transform Infrared Spectroscopy

ATR‐FTIR spectra of the samples were recorded on a VERTEX‐80v Fourier transform spectrometer (Bruker, Germany) in the 4000–400 cm^−1^ range at a spectral resolution of 4 cm^−1^. Spectra were processed using the OPUS software package.

The hydrocarbon and carbonyl indices, as well as their degrees of change, were calculated.

The hydrocarbon index (*I*_*h*_) was calculated using Formula (1):

(1) Ih=HCHHCN

where *H*_*C* *H*_—3060–2716 a.u. peak area and *H*_*C* *N*_—2271–2209 a.u. peak area.

The carbonyl index (*I*_*c*_) was calculated using Formula (2):

(2) Ic=HCOHCN,

where *H*_*C* *O*_—1807–1492 a.u. peak area.

The degree of change in the indices was calculated using the Formula (3):

(3) A=ISmplIContr,

where *I*_*S* *m* *p* *l*_ is the hydrocarbon or carbonyl index of the NBR after exposure, *I*_*C* *o* *n* *t* *r*_ is the hydrocarbon or carbonyl index of the NBR without treatment.

### 2.6. Statistical Analysis

Statistical data processing was performed using GraphPad Prism 8.0.1. Results were obtained from at least three independent experiments. The mean, standard deviation, and standard error of the mean were determined. The data were tested for normality, and the significance of differences was analyzed using one‐way ANOVA, *p* < 0.05 with post hoc Tukey′s HSD test.

## 3. Results

### 3.1. Growth of *R. qingshengii* IEGM 1416 and *R. erythropolis* IEGM 1417 on Mineral Medium With Various Nitrogen and Carbon Sources

**Figure 1:**
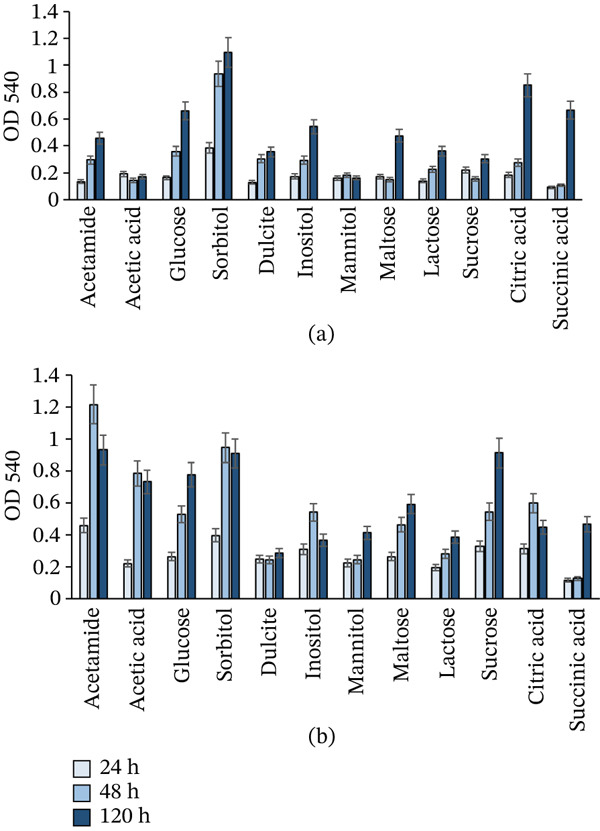
The growth of (a) *R. qingshengii* IEGM 1416 and (b) *R. erythropolis* IEGM 1417 on a mineral medium supplemented with various carbon sources.

*Rhodococcus* strains were grown on a mineral medium supplemented with various carbon sources. The optimal carbon source was identified based on biomass accumulation. *R. qingshengii* IEGM 1416 and *R. erythropolis* IEGM 1417 exhibited the highest growth rates on sorbitol and acetamide, respectively (Figure 1).

**Figure 2:**
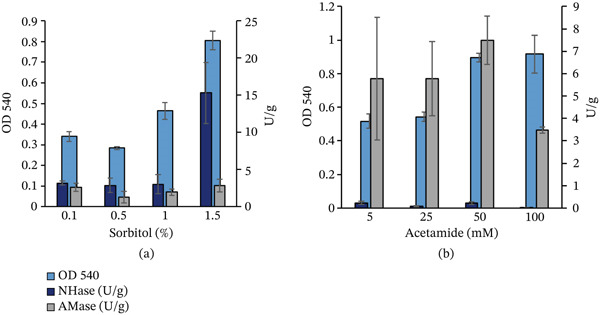
The effect of sorbitol and acetamide concentrations on the growth (OD_540_), nitrile hydratase, and amidase activities (U/g) of (a) *R. qingshengii* IEGM 1416 and (b) *R. erythropolis* IEGM 1417.

The optimal sorbitol and acetamide concentrations for biomass accumulation and the manifestation of nitrile hydratase and amidase activities in *R. qingshengii* IEGM 1416 and *R. erythropolis* IEGM 1417 were determined. It was shown that the amidase activity of *R. qingshengii* IEGM 1416 was independent of sorbitol concentration and remained relatively low, whereas its nitrile hydratase activity significantly exceeded that of *R. erythropolis* IEGM 1417 and was maximal at 1.5% sorbitol (Figure 2). Moreover, biomass accumulation was highest in the medium containing 1.5% sorbitol. *R. erythropolis* IEGM 1417 accumulated more biomass in media with 50 and 100 mM acetamide; however, its amidase activity was maximal at 50 mM acetamide. Therefore, to optimize biomass accumulation, *R. qingshengii* IEGM 1416 and *R. erythropolis* IEGM 1417 were grown on mineral medium with 1.5% sorbitol and 50 mM acetamide, respectively.

**Figure 3:**
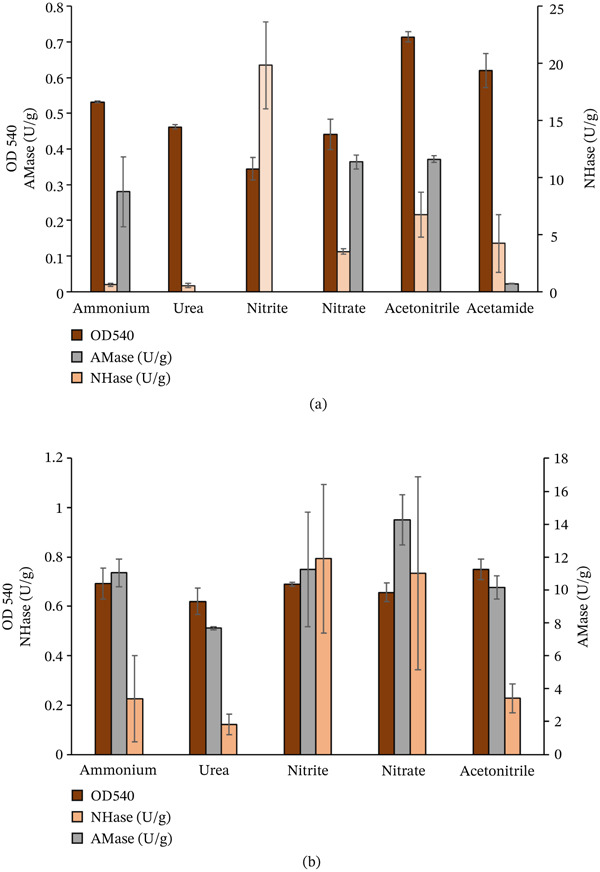
The growth of (a) *R. qingshengii* IEGM 1416 and (b) *R. erythropolis* IEGM 1417 on different nitrogen sources.

The optimal nitrogen sources for the growth and nitrile hydratase activity of *R. qingshengii* IEGM 1416 and *R. erythropolis* IEGM 1417 were determined. It was shown that the growth of *R. qingshengii* IEGM 1416 was maximal with acetonitrile as the sole nitrogen source, whereas the strain exhibited the highest nitrile hydratase activity when grown on sodium nitrite (Figure 3a). The amidase activity of this strain was suppressed during growth on nitrite; similarly, both nitrile hydratase and amidase activities were inhibited when urea was used as the nitrogen source.

Nitrile hydratase activity of *R. qingshengii* IEGM 1416 exceeds amidase activity, reaching 20 U/g, whereas amidase activity of *R. erythropolis* IEGM 1417, conversely, is significantly higher than nitrile hydratase activity, reaching a maximum (14 U/g) with nitrate (Figure 3b). Accumulation of *R. erythropolis* IEGM 1417 biomass is independent of the nitrogen source. Nitrites and nitrates induce equal levels of activity for both nitrile hydratase and amidase.

**Figure 4:**
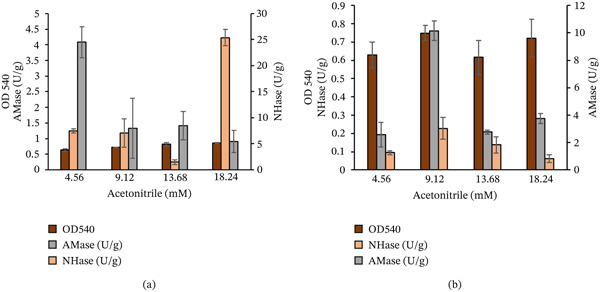
The effect of different concentrations of acetonitrile on the growth and nitrile hydratase and amidase activities of (a) *R. qingshengii* IEGM 1416 and (b) *R. erythropolis* IEGM 1417.

Acetonitrile is a selective nitrogen source for bacteria harboring nitrile‐hydrolyzing enzymes. The presence of acetonitrile resulted in the expression of both nitrile hydratase and amidase activities, although neither was maximal. The effect of various acetonitrile concentrations on the growth and nitrile‐hydrolyzing activity of *Rhodococcus* strains was further studied. It was shown that the nitrile hydratase and amidase activities of *R. qingshengii* IEGM 1416 were maximal at 18.24 and 4.56 mM acetonitrile, respectively, whereas acetonitrile concentration had no effect on biomass accumulation (Figure 4a). The maximum amidase activity of *R. erythropolis* IEGM 1417 was observed at 9.12 mM acetonitrile (Figure 4b).

### 3.2. Growth of *R. qingshengii* IEGM 1416 and *R. erythropolis* IEGM 1417 Using NBR as the Sole Nitrogen Source

Growth of *R. qingshengii* IEGM 1416 and *R. erythropolis* IEGM 1417 on a medium containing NBR as the sole nitrogen source was studied. The concentration of acrylonitrile and its transformation products (acrylamide and acrylic acid) in the medium was determined after cultivating *Rhodococcus* strains with NBR. Acrylamide was not detected in any of the samples. Nitrile‐utilizing bacteria harboring nitrile hydratase and amidase are known to use acrylamide as a sole carbon source for biomass accumulation [18]. Consequently, acrylamide formed during acrylonitrile transformation is integrated into the metabolic pathways of these bacteria. Acrylonitrile and acrylic acid were present in the liquid phase after UV exposure of NBR. Additionally, acrylonitrile was present in the samples after cultivation of *R. erythropolis* IEGM 1417. The amount of acrylic acid did not exceed 0.06 mg/L, and the acrylonitrile concentration ranged from 5 to 9 mg/L. In the other samples, including those after cultivation of *R. qingshengii* IEGM 1416, acrylonitrile monomers were not detected; this could be associated with the more active growth of this strain, utilizing them as a carbon and energy source.

**Figure 5:**
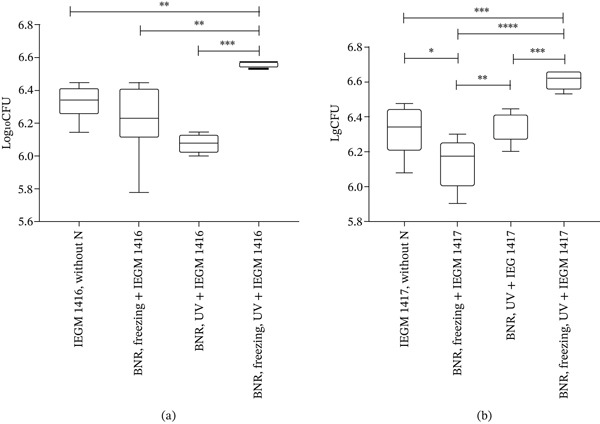
CFU counts of (a) *R. qingshengii* IEGM 1416 and (b) *R. erythropolis* IEGM 1417 in the culture medium after growth with NBR pretreated with UV and freeze‐thawing (ANOVA with post hoc Tukey′s HSD test, ∗*p* = 0.02, ∗∗*p* = 0.005, ∗∗∗*p* = 0.0004, ∗∗∗∗*p* < 0.0001).

The number of CFU/mL in the culture media was determined after 2 weeks of cultivation with NBR (Figure 5a,b). When *Rhodococcus* strains were cultured with NBR pretreated with UV radiation and freeze‐thawing, the number of CFU/mL was statistically significantly higher (*p* < 0.05) than after cultivation without a nitrogen source.

After growth of *R. qingshengii* IEGM 1416 and *R. erythropolis* IEGM 1417 with NBR for 1 month without an additional nitrogen source, the number of viable cells in the biofilm was determined. The biofilms of *R. qingshengii* IEGM 1416 and *R. erythropolis* IEGM 1417 grown on treated NBR contained (7.28 ± 0.48) × 10^6^ and (6.27 ± 0.72) × 10^6^ CFU/cm^2^, respectively. Thus, pretreatment of NBR with UV combined with freeze‐thawing promotes the formation of a robust biofilm.

### 3.3. Scanning Electron Microscopy of *Rhodococcus* Biofilms on the NBR Surface

**Figure 6:**
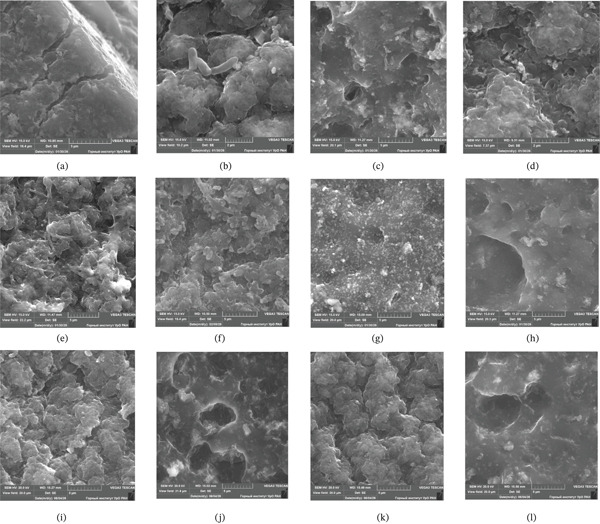
Scanning electron microscopy of NBR. The polymer was pretreated with (a, c, e, f) UV and freeze‐thawing or (b, d) UV only. Micrographs show biofilms of (a, b, e) *R. qingshengii* IEGM 1416 and (c, d, f) *R. erythropolis* IEGM 1417 grown on the polymer for (a–d) 14 days and (e, f) 1 month. Controls include: (g) untreated polymer without bacterial growth and without incubation in mineral medium; (h) UV‐treated polymer without bacterial growth and without incubation in a mineral medium; UV‐treated polymer after incubation in a sterile mineral medium, (i) rough side and (j) smooth side; UV‐ and freeze‐thawing treated polymer after incubation in a sterile mineral medium, both sides: (k) rough and (l) smooth.

Micrographs of *R. qingshengii* IEGM 1416 and *R. erythropolis* IEGM 1417 biofilms on NBR were obtained (Figure 6). The NBR had two distinct sides: a rough surface (Figure 6i,k) and a smooth porous surface (Figure 6h, j, and l), which resulted in uneven biofilm growth. However, NBR surfaces did not differ significantly when treated with UV only, UV and freeze‐thawing, or after incubation of the pretreated polymer in a sterile mineral medium (Figure 6h–l). Micrographs showed that the biofilm grew actively on the rough surface of the polymer (Figure 6a, b, and d–f). At the same time, biofilms also formed on the smooth surface, but to a lesser extent (Figure 6c). A dense biofilm formed on the NBR after 1 month of cultivation following inoculation with a large volume of biomass (Figure 6e,f). The biofilm consisted of a mucoid matrix with rod‐shaped cells embedded deep within the EPS. The outlines of elongated cells were clearly visible in the structure of the *R. qingshengii* IEGM 1416 and *R. erythropolis* IEGM 1417 biofilms. A mucoid polymer matrix with less distinct cell outlines was observed after 2 weeks of growth on NBR (Figure 6a–d); in some cases, rod‐shaped cells were noted on the surface, outside the EPS (Figure 6b).

### 3.4. Evaluation of Structural Changes in NBR Mediated by *Rhodococcus* Strains Using Fourier Transform Infrared Spectroscopy

**Figure 7:**
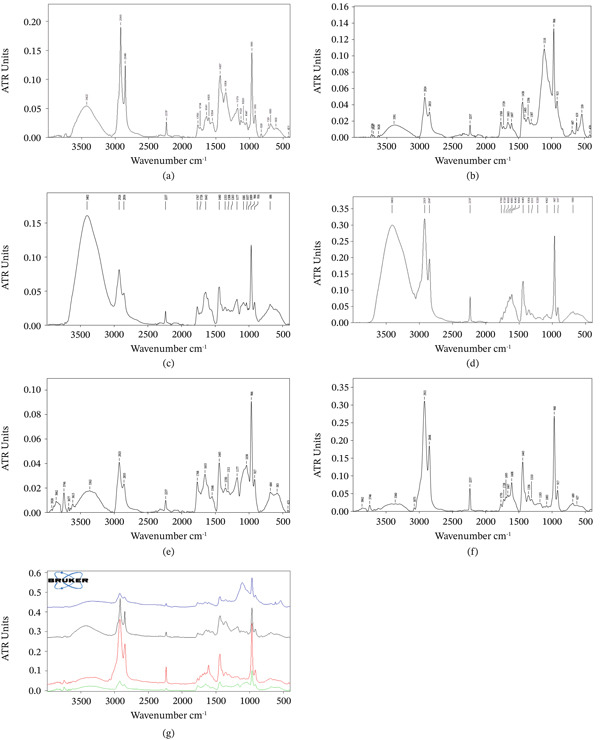
ATR‐FTIR spectra of NBR: (a) untreated NBR; (b) NBR after UV treatment and freeze‐thawing; NBR after UV treatment, freeze‐thawing and after incubation in a sterile mineral medium, (с) rough side and (d) smooth side; NBR after growth of *R. qingshengii* IEGM 1416 for 1 month without an additional nitrogen source on (e) rough side and (f) smooth side of the pretreated polymer. (g) Combined spectra: black line—untreated NBR; blue line—NBR after UV treatment and freeze‐thawing; green and red lines—NBR after growth of *R. qingshengii* IEGM 1416 on rough side and smooth side, respectively.

ATR‐FTIR spectra were obtained for untreated NBR, after UV exposure and freeze‐thawing, after incubation of UV‐treated and freeze‐thawed BNR in a sterile mineral medium, and after growth of *R. qingshengii* IEGM 1416 for 1 month on NBR pretreated with UV and freeze‐thawing without an additional nitrogen source (Figure 7).

The peak heights of the samples were compared (Table 1). ATR‐FTIR spectra confirm that the material is NBR [19, 20]. All samples exhibited a stable peak characteristic of the nitrile group (2237 cm^−1^) and an intense peak at 966 cm^−1^, typical of the deformation vibrations of trans‐C=C bonds indicative of butadiene units.

**Table 1:** Relative intensities of the ATR‐FTIR absorption peaks for NBR samples.

Peak, cm^−1^	Chemical group	NBR without incubation in mineral medium		NBR after UV treatment, freeze‐thawing, and incubation in mineral medium			
		Without treatment	After UV treatment and freeze‐thawing	Sterile		Bacterial growth	
				Rough	Smooth	Rough	Smooth
Peak intensity, a.u.							
2919–2929	C–H bonds (aliphatic groups)	0.179	0.070	0.072	0.195	0.048	0.327
2850–2866	C–H bonds (aliphatic groups)	0.111	0.051			0.03	0.201
2237	Nitrile	0.023	0.019	0.020	0.077	0.011	0.066
1769	Ester plasticizers		0.024	0.026			
1729	Carbonyl group		0.019				
1667	Amide groups Amide I	n.d.	n.d.	n.d.	n.d.	n.d.	0.050
1642–1663	Stretching vibrations of C=C bonds	0.038	0.025	0.046			
1653						0.033	
1606–1607	Aromatic rings (presence of plasticizers)	0.036	0.024				0.092
1548–1551	Amide groups Amide II	n.d.	n.d.	n.d.	n.d.	0.016	n.d.
1438–1443	Deformation vibrations of CH_2_ groups located next to the nitrile group	0.11	0.053	0.052	0.113	0.045	0.153
1366	Deformation vibrations of CH_3_ groups		0.037				
1354	Deformation symmetric vibrations of C–H bonds in methyl groups (−CH_3_)	0.081					
1110	C–O or C–O–C bonds (ethers)		0.125				
966–967	Deformation vibrations of C=C bonds	0.138	0.150	0.111	0.257	0.095	0.280

Abbreviation: n.d., not detected.

It is known that polymer aging causes surface changes (e.g., cracks and roughness) and alterations in ATR‐FTIR spectra [21]. A comparison of the peaks (Figure 7) reveals that the treated samples, in contrast to the original material, exhibit a decrease in the content of hydroxyl/amine groups, the disappearance of the broad plateau at 3422 cm^−1^, and a redistribution in the carbonyl region (1730–1770 cm^−1^), which often accompanies the aging or chemical modification of polymers. The low spectral intensity and changes in the 1730–1770 cm^−1^ region confirm oxidation under the influence of UV light. The different intensities (0.10 versus 0.35) of the peaks for the NBR samples after incubation with *Rhodococcus* strains indicate surface differences, which may be associated with varying roughness and, consequently, uneven biofilm accumulation. A change in the carbonyl region (1700–1800 cm^−1^) of the UV‐exposed samples is observed, specifically the appearance of peaks at 1769 and 1729 cm^−1^, indicating the formation of new oxygen‐containing groups (ketones, aldehydes, or esters) resulting from polymer chain scission and oxygen addition under the influence of light. The signal amplitude decreases, which is a characteristic sign of photoaging. However, the nitrile peaks are preserved, as the acrylonitrile units of the copolymer are more resistant to UV exposure than the butadiene regions or labile impurities.

Peaks in the ATR‐FTIR spectra in the range of 1630–1690 cm^−1^ and 1530–1560 cm^−1^ indicate the presence of amide groups—Amide I and Amide II, respectively [22, 23]. The appearance of new peaks in the 1548–1667 cm^−1^ region, characteristic of amide groups in the sample spectra after bacterial treatment, confirms the transformation of nitriles into amides catalyzed by nitrile hydratases. On the smooth side of the polymer, a peak (0.050) is observed at 1667 cm^−1^, confirming the conversion of a nitrile (–C≡N) to an amide group (–CONH_2_). The peak at 1608 cm^−1^ may indicate the formation of double bonds conjugated with carbonyls or the accumulation of intermediate oxidation products. The difference in the set of peaks and their intensities between sides confirms that bacterial growth occurred unevenly. Oxidative aging is confirmed by the appearance of carbonyl groups (1729 cm^−1^) and amide groups (1667, 1548–1551 cm^−1^) relative to the unchanged groups, such as nitrile (2237 cm^−1^) or aliphatic C–H (2919–2924 cm^−1^).

It has been established that amide groups do not form during UV exposure, freeze‐thawing, and after incubation of treated polymer in sterile mineral medium. Growth of *Rhodococcus* strains results in the accumulation of amide compounds, indicating changes in the polymer chain under the influence of microbial enzymes.

Significant changes in the peak areas corresponding to various chemical groups are observed (Table 2). The peak area at 2919–2850 cm^−1^, corresponding to hydrocarbon groups, decreases after UV treatment (from 17.944 to 13.131 a.u.) and decreases significantly after bacterial growth on rough side (to 6.627 a.u.) compared with the original sample. Moreover, incubation of the UV‐treated and freeze‐thawed polymer in a sterile mineral medium leads to a decrease in the integrated peak areas (to 8.384 a.u.), but remains higher than that of the BNR after bacterial growth. This may indicate modification of the hydrocarbon base of the rubber under the influence of UV radiation, incubation in a sterile mineral medium, especially following exposure to *Rhodococcus* strains. The area of the peak at 2237 cm^−1^, corresponding to the nitrile group, decreases after UV treatment (from 0.645 to 0.567 a.u.), after treatment and incubation in a sterile mineral medium (to 0.442 a.u.) and after bacterial growth on rough side (to 0.379 a.u.), indicating chemical changes or degradation of nitrile groups in the polymer structure. The area of the peak at 1807–1492 cm^−1^ decreases slightly after UV exposure and incubation in a sterile mineral medium (from 6.931 to 6.662 a.u.), and decreases after bacterial growth on rough side (to 5.892 a.u.). The appearance or strengthening of peaks in this region (around 1700 cm^−1^) is often associated with the formation of carbonyl groups (C=O) as a result of oxidation or other degradation reactions, which is typical of polymer aging processes under external influences. Changes observed in the fingerprint region (below 1500 cm^−1^) indicate chemical transformations of the plasticizers present in the original sample (peaks at 1182, 1127, 1092, 1047 cm^−1^).

**Table 2:** Integrated peak areas of the ATR‐FTIR spectra for NBR samples.

Peak, cm^−1^	Chemical group	NBR without incubation in mineral medium		NBR after UV treatment, freeze‐thawing, and incubation in mineral medium			
				Sterile		Bacterial growth	
		Without treatment	After UV treatment and freeze‐thawing	Rough	Smooth	Rough	Smooth
Peak area, a.u.							
3060–2716	Hydrocarbon groups	17.944	13.131	8.384	34.585	6.627	38.114
2271–2209	Nitrile group	0.645	0.567	0.442	1.258	0.379	1.370
1807–1492	Carbonyl groups	6.931	5.388	6.662	11.126	5.892	12.480
1492–848	—	29.951	38.646	14.126	17.865	18.351	32.668

The hydrocarbon index, reflecting the total proportion of the hydrocarbon skeleton (both saturated and partially unsaturated) relative to the polar units of acrylonitrile, and the carbonyl index, indicating the degree of oxidation of the hydrocarbon skeleton, were calculated (Table 3). It was found that the hydrocarbon index consistently decreases with UV irradiation, incubation in a sterile medium, and bacterial growth, whereas the carbonyl index increases during both sterile medium incubation and *Rhodococcus* cultivation.

**Table 3:** Hydrocarbon and carbonyl indices of NBR.

Sample		Hydrocarbon index/A	Carbonyl index/A
NBR without incubation in mineral medium	Without treatment	27.82/1	10.75/1
	After UV treatment and freeze‐thawing	23.16/0.83	9.50/0.88
NBR after UV treatment, freeze‐thawing, and incubation in mineral medium	Sterile	18.97/0.68	15.07/1.40
	Bacterial growth	17.49/0.63	15.55/1.45

## 4. Discussion

The processes of biocorrosion and biodegradation of polymers are associated with the ability of microorganisms to form biofilms [24], with enhanced microbial growth occurring in areas already damaged by abiotic environmental factors [25]. Biofouling is the first stage of material degradation. Bacterial colonization of the surface creates microstresses that promote the formation of microcracks, facilitating the penetration of oxygen and enzymes into the material and accelerating chemical degradation. Since NBR contains butadiene and acrylonitrile units, the biodegradation of this polymer requires microorganisms capable of degrading both the butadiene component (hydrocarbon backbone) and the acrylonitrile units.

Among the bacteria that degrade rubber, taxa containing mycolic acids in their cell walls (*Gordonia*, *Nocardia*, and *Mycobacterium*) are the most common. The biodegradation process is limited to the rubber surface due to its structural complexity and insolubility in water [26]. A major obstacle to the microbial degradation of plastics is their hydrophobicity, which hinders cell contact with the surface. The production of biosurfactants by bacteria is a key factor in degradation, as these molecules reduce surface tension and facilitate bacterial attachment [27]. Actinobacteria of the genus *Rhodococcus* are capable of degrading various xenobiotics, synthesizing a wide range of degradative enzymes—such as dehydrogenases, peroxidases, oxygenases, alkyl sulfatases, and phenol hydrolases—and producing glycolipid biosurfactants [28]. We previously isolated two *Rhodococcus* strains from an extreme biotope of anthropogenic origin that combined polyextremotolerance (resistance to high pH and salinity) with the ability to hydrolyze nitriles [17]. In this study, we investigated the ability of these strains to grow with NBR as the sole nitrogen source, their biofilm formation on NBR, and their effect on the polymer structure.

The degradation of NBR by microorganisms is a complex, multistage process affecting both the polymer backbone and the functional groups. Key changes in the polymer proceed via several pathways: first, microbial oxygenases and peroxidases attack the double bonds of the butadiene units (peaks at 966–967 cm^−1^), resulting in chain scission and the formation of shorter fragments. New oxygen‐containing groups (aldehydes, ketones, and carboxylic acids) appear at the scission sites, explaining the changes in the 1600–1750 cm^−1^ region. Based on the hydrocarbon and carbonyl indices calculations, it can be concluded that hydrocarbon chains undergo partial degradation under the influence of UV and bacterial enzymes; their intensity decreases, whereas the carbonyl index increases, indicating hydrocarbon oxidation. The significant decrease in the nitrile peak height (2237 cm^−1^) on the rough side of NBR upon exposure to bacteria, accompanied by a slight decrease in the peak height of butadiene units (966–967 cm^−1^), indicates that bacteria primarily utilize nitrile groups. This is consistent with the fact that *Rhodococcus* strains grew in the presence of a carbon source, but with NBR as the sole nitrogen source, eliminating the need to use the polymer′s carbon backbone as a growth substrate.

Hydrolysis of nitrile groups occurs via nitrilases or nitrile hydratases: nitriles are transformed into carboxylic acids by nitrilases, or into amides by nitrile hydratases, with the latter further hydrolyzed into carboxylic acids by amidases. The appearance of new peaks in the 1548–1667 cm^−1^ region, characteristic of amide groups, confirms this process. We previously confirmed that *R. qingshengii* IEGM 1416 and *R. erythropolis* IEGM 1417 harbor genes encoding iron‐containing nitrile hydratases and amidases [29] and exhibit nitrile hydratase activity even in alkaline media [17]. Intense peaks at 1667 and 1551 cm^−1^, appearing in ATR‐FTIR spectra of NBR during the growth of nitrile‐utilizing rhodococci, provide evidence of biochemical degradation that is not observed with UV treatment alone. A limitation of this study is the short observation period (2 weeks to 1 month), which cannot provide comprehensive data on the degradation of NBR. We conclude that initial changes occurring on the NBR surface are caused by a combination of physical factors (UV, freezing and thawing, incubation in a mineral medium) and biological processes (the growth of a biofilm of nitrile‐utilizing bacteria on the polymer surface).

Microorganisms often initiate the degradation not of the polymer itself, but of low‐molecular–weight additives (plasticizers and antioxidants), which are more easily assimilated. Changes in the region below 1500 cm^−1^ indicate chemical alterations in the plasticizers present in the original sample. Plastic degradation under natural conditions is complex and involves a combination of photodegradation and biodegradation; the extent of photodegradation, which precedes biodegradation, is significantly dependent on the types of additives used [30]. NBR, the primary component of nitrile gloves, may contain residual vulcanization accelerators, antioxidants, or related substances capable of releasing trace amounts of nitrogen. Following UV irradiation, freeze‐thaw cycling, and prolonged incubation of NBR in a nitrogen‐free medium, ammonium ion concentrations were 2 mg/L. Although insufficient to support sustained bacterial growth as a sole nitrogen source, this trace amount may facilitate initial bacterial proliferation and enhance early‐stage biofilm formation on NBR.

Nitrile‐hydrolyzing enzymes are intracellular; however, they can be released into the environment upon cell lysis. A biofilm is a complex community containing living, actively dividing cells, as well as persistent and dead cells. The EPS matrix is an essential component of this architecture. This matrix not only promotes cell adhesion and initiates the biocorrosion process, but also provides a protective environment and a reservoir for extracellular substances, exoenzymes, including enzymes released from lysed cells. Consequently, the retention of these stable enzymes effectively renders the biofilm matrix into an external digestion system [31].

## 5. Conclusions

The haloalkalitolerant *R. qingshengii* IEGM 1416 and *R. erythropolis* IEGM 1417 are capable of biofilm formation on NBR in the absence of an additional nitrogen source. When grown with NBR as the sole nitrogen source, *R. qingshengii* IEGM 1416 alters the polymer structure. To increase the efficiency of this process, UV radiation and freeze‐thawing pretreatment of NBR are necessary. The appearance of amide groups in the polymer structure after the growth of nitrile‐utilizing rhodococci was established, resulting from the transformation of nitrile groups by the nitrile hydratases of these bacteria. Oxidative aging of the material was observed after both UV irradiation and bacterial growth. Biofilm formation was confirmed by both scanning electron microscopy and direct CFU counting. Enzymes from haloalkalitolerant nitrile‐utilizing rhodococci induce chemical changes in NBR, corresponding to the initial stages of backbone oxidation and nitrile group transformation.

## Author Contributions

G.A.S. performed experiments, analyzed the data, and wrote the first draft of the manuscript. A.Y.M. was involved in data analysis and corrected the manuscript. O.V.K. performed electron microscopy of samples. Y.G.M. conceived and designed the study and wrote the final version of the manuscript.

## Funding

No funding was received for this manuscript.

## Disclosure

The study was carried out within the framework of the state assignment on the topic “Biodiversity of microorganisms in anthropogenically polluted ecosystems and functional genetic mechanisms of their adaptation to stressful environmental conditions,” Registration Number 124020500028‐4. All the authors have read and approved the final manuscript.

## Conflicts of Interest

The authors declare no conflicts of interest.

## Data Availability

The data that support the findings of this study are available from the corresponding author upon reasonable request.
